# Compression may be a general tendency of auditory distance judgments: evidence from distance estimates using a novel echolocation skill

**DOI:** 10.1007/s00221-025-07180-y

**Published:** 2025-10-21

**Authors:** Andrew J. Kolarik, Samuel Evans, Eleanor McCarthy, Brian C. J. Moore

**Affiliations:** 1 School of Psychology, University of East Anglia, Norwich, UK; 2 Cambridge Hearing Group, Department of Psychology, University of Cambridge, Cambridge, UK; 3 Vision and Eye Research Institute, Faculty of Health, Education, Medicine and Social Care, Anglia Ruskin University, Cambridge, UK; 4 Research and Development, Norfolk and Suffolk NHS Foundation Trust, Norwich, UK

**Keywords:** Localization, Spatial hearing, Auditory distance, Echolocation, Depth

## Abstract

Estimates of sound source distance are well described by a compressive function relating judged to actual distance; distance is systematically underestimated at larger distances. The current experiment investigated whether such compression is a general tendency of auditory distance judgments, by measuring the judged distance of silent objects based on a novel skill, using self-produced echolocation mouth clicks. The accuracy and precision of distance estimates were measured for aluminum or foam objects (the latter being less reflective than the former) positioned 30, 60, or 90 cm away from 11 blindfolded, normally sighted participants. The distance estimates were well characterized by compressive power functions. Distances were significantly more underestimated and consistency was significantly worse for the two closest object distances for foam than for aluminum objects. Systematic errors were similar for the two materials. The results are consistent with the idea that compression may be a general tendency of auditory distance judgments, both for sound-producing objects as observed in the literature and for silent objects whose distance is judged using a novel echolocation skill.

## Introduction

An established general characteristic of auditory distance judgments for sound-producing objects, that is distinct from other aspects of spatial perception such as azimuth or elevation judgments, is that judged distance is a compressive power function of actual distance; the sound source distance is systematically underestimated as sound source distance increases (Zahorik et al. 2005). This has been observed across a wide range of acoustic environments and cue conditions (for reviews, see Zahorik et al. 2005; Kolarik et al. 2016a). It may reflect the fact that the cues for distance have a compressive characteristic, changing more slowly with distance as distance increases. Underestimation of farther source distances may result from linear mapping of the available auditory cues to the judged distance of the sound source, whereas accurate distance estimates would require an expansive mapping. In general, relevant cues to distance, most prominently sound level (Coleman 1963; Mershon and King 1975) and direct-to-reverberant energy ratio (Bronkhorst and Houtgast 1999; Zahorik 2002b), have high values for nearby sound sources, and decrease monotonically as distance increases, allowing a monotonic mapping between cue magnitude and judged distance. However, beyond a certain source distance, auditory cues to distance may remain effectively constant, for example when the reverberant sound has a much higher level than the direct sound, and an “auditory horizon” is reached (Bronkhorst and Houtgast 1999) making discrimination of changes in distance more difficult or impossible (Larsen et al. 2008). As noted above, the compression of judged distance may be a consequence of participants not allowing adequately for the way that the effectiveness of distance cues changes with distance or it may reflect a bias to judge a sound as closer when the available cues do allow reliable distance estimates.

If compression is a general tendency of auditory distance judgments, then a compressive power function should be evident for distance judgments based on an auditory skill with which participants have no prior experience, such as echolocation. The magnitude of the sound echoes, and hence the strength of the available echolocation cues at the listener’s ears, decreases with increasing distance for a reflective object of fixed size (Papadopoulos et al. 2011). The specific cues used, and the corresponding perceptual attributes, may vary with distance. An analysis of the results of Schenkman and Nilsson (2010) by Schenkman and Gidla (2020) suggested that for smaller distances participants appeared to use repetition pitch (the pitch heard when a delayed version of a sound is added to the original sound, Bilsen 1966) and loudness cues. The salience of repetition pitch decreases with increasing distance, because salience reduces as the level of the reflected sound relative to the emitted sound decreases and as the delay increases (Yost et al. 1996). Loudness cues also become less effective with increasing object distance, partly because level discrimination worsens at low levels (Florentine et al. 1987). For farther distances Schenkman and Gidla (2020) suggested that aspects of timbre such as sharpness were used. These changes in timbre result partly from the fact that the absorption of sound by transmission though air is greater at high than at low frequencies. All of the available echolocation cues change more slowly with distance as distance increases. In other words, the mapping of cue effectiveness to distance is compressive.

Since the available distance cues are compressive in nature for both silent objects using cues derived from self-produced echolocation clicks and for sound-producing objects, it is likely that echolocation distance estimates will also be underestimated at farther distances, stemming from a general inability to compensate for compressive cues. The current experiment investigates this.

Echolocation has been demonstrated to support effective navigation and perception of the environment in the absence of visual information (for reviews, see Stoffregen and Pittenger 1995; Kolarik et al. 2014; Thaler and Goodale 2016). Sound echoes from self-generated noises, such as mouth clicks or cane taps, are used to construct representations of physical space (Flanagin et al. 2017; Norman et al. 2021).

Previous work has demonstrated better echolocation performance for blind individuals than for normally sighted controls, for tasks such as obstacle circumvention (Kolarik et al. 2017b) or assessing whether or not a reflecting disc was placed in front of an acoustic manikin (Schenkman and Nilsson 2010) or artificial head (Schenkman and Nilsson 2011). Enhanced echolocation abilities are likely the result of the beneficial effects of crossmodal brain plasticity following vision loss (Kolarik et al. 2021), or practice (Norman et al. 2021), or a combination of the two.

It has been shown that echolocation can be used to discriminate object distance. Kellogg (1962) presented a sound-reflective disc coated in sand-texture paint at a standard distance and at a comparison distance that was closer or farther than the standard in random order. Participants were asked to use self-generated sounds to report which disc was closer. While blind participants were able to discriminate distances, blindfolded sighted participants did not perform significantly above chance. The blind participants performed the task using clicking, finger snapping and hissing sounds, or, more often, vocal sounds. Schörnich et al. (2012) showed that following extensive training lasting between 4 and 12 weeks, sighted participants were able to discriminate object distances using echolocation. For a reference distance of 1.7 m, just-noticeable-differences were often lower than half a meter, corresponding to a Weber fraction of 0.29. This is comparable to the Weber fraction found by Kellogg (1962) for blind participants, which was between 0.18 and 0.30.

Tonelli et al. (2016) showed that sighted, untrained individuals could learn to use echolocation to judge the relative distances of single plexiglass objects, from a set of 5 distances. Participants were presented with a vertical bar on each trial, at distances ranging from 30 to 150 cm in 30-cm steps. They were instructed to use mouth- or hand-generated clicks to judge the bar distance, verbally reporting a number from 1 to 5, where 1 was the closest distance and 5 the farthest. Feedback was provided. The accuracy of the judgments increased over sessions and was significantly better in a reverberant than in an anechoic testing room for the closest test distance only. There was no significant effect of type of echolocation sound (mouth clicks or finger snaps). However, it is unknown how well sighted participants can use echolocation to judge absolute distance.

The effectiveness of echolocation depends on the sound-reflective properties of the target object. Hard materials such as concrete reflect sound over a wide frequency range, whereas soft materials such as carpet tend to reflect only low-frequency sound (Stoffregen and Pittenger 1995). Changes in the spectrum of the sound at the participant’s ears resulting from the addition of the echo to the emitted sound provide an echolocation cue (Kolarik et al. 2014). Hence, a decrease in the available echo frequencies for soft materials may result in poorer spatial judgments as well as less accurate judgments of the properties of the objects. Hausfeld et al. (1982) showed that untrained sighted participants were able to use echolocation to recognize fabric and wooden discs, but not those composed of carpet or Plexiglas, from the selection of materials available. It is surprising that performance was not better for Plexiglas, as it is reflective over a wide frequency range. However, the authors highlighted the tendency for participants to report Plexiglas as wood, suggesting that wood and plexiglass may be confusable in a recognition task due to the high reflectivity of both materials. The effect of material on echolocation absolute distance judgments (which would not involve distinguishing between different object materials) has not yet been investigated.

The current study assessed the ability of blindfolded sighted participants to use echolocation to judge the distance of objects that were more reflective (aluminum) or less reflective (foam). The primary motivation for the study was to provide insight regarding how people use novel cues to construct internal spatial representations of distance, and to assess whether distance judgments for farther silent objects based on these novel cues are underestimated, as for sound-producing sources. If the distance judgments are systematically underestimated as object distance increases, this would support the possibility that compressive mapping is a general phenomenon. The findings were also intended to be valuable in evaluating the extent to which people who suddenly lose their sight might be able to use echolocation for estimating object distance and evaluating how the accuracy of such judgments might be affected by object material. It was hypothesized that judged echolocation distance would be a compressive function of actual distance (Zahorik et al. 2005). Higher object reflectivity increases the range of frequencies reflected by the object (Stoffregen and Pittenger 1995), which may provide more salient echolocation cues. Hence, it was also hypothesized that echolocation distance judgments would be significantly more accurate and more consistent for an aluminum-covered object than for a less reflective foam-covered object.

## Methods

There were a number of methodological differences between the present experiment and that of Tonelli et al. (2016), which also assessed the ability to judge distance using echolocation. Tonelli et al. (2016) trained participants using feedback for a set of object test distances that was also used in the main experiment, and an object was always present on each trial. Participants were not required to report absolute distances, but instead reported a number from 1 to 5 where where 1 was the nearest test distance and 5 the farthest, for vertical bars where the angle subtended by the bars at the participant was kept constant over the test distances. Bars that were farther way were longer than bars that were nearby. Tonelli et al. (2016) reported that they did this to prevent judgments of distance based on the angle subtended by the bars. The current experiment assessed absolute distance judgments for objects of the same size presented at different distances. This represents a more ecologically valid scenario, as objects do not change size with distance. Also, “catch” trials were included in which no object was present (Ashmead et al. 1989; Kolarik et al. 2016b, 2017b). This reflects a situation where an individual might use echolocation to assess whether or not an object is present in a room, and, if an object is judged to be present, to use its location as a waypoint during navigation.

### Participants

Eleven participants (5 females, mean age 19.3 years, range 18–20 years) were tested. All were students recruited internally from the University of East Anglia who took part for course credits. All participants self-reported normal or corrected to normal vision and no hearing impairment, and all had no prior echolocation experience. Participants’ pure tone thresholds were less than or equal to 25 dB HL for all audiometric frequencies up to 8 kHz, measured with an Interacoustics AS608 audiometer using methods described by the British Society of Audiology (2011). All participants provided written informed consent, and the study was approved by the University of East Anglia’s Ethics Subcommittee and conducted in line with the Declaration of Helsinki.

### Apparatus and data acquisition

Each participant was tested in the center of a quiet laboratory measuring 21 m ×  8 m ×  3 m with an ambient sound level of approximately 36 dBA. The room was carpeted, with plastered walls. A rectangular cork board (90 × 60 cm) was presented on each trial (except for catch trials). In the familiarization phase, the board was uncovered (i.e. participants were presented with cork, for which the reflectiveness was intermediate between those for the two conditions with the board covered) and in the main experiment the board was covered with either aluminum foil or foam. A rectangular object covered in aluminum was chosen following the use of such an object in an echolocation shape-determination study (Milne et al. 2014) and in experiments that investigated the effect of object distance on echolocation detection abilities (Schenkman and Nilsson 2010, 2011), and because aluminum was more reflective than foam. The boards were attached to long, lightweight metal rods, which could be readily adjusted in height and moved according to the marked distances. The boards were presented in landscape orientation directly facing the participant. Each board was adjusted so that its centre height was the same as the height of the participant’s mouth, and participants were instructed to scan the object using echolocation clicks, following Tonelli et al. (2016). Participants were blindfolded throughout the experiment and were informed when it was time to start each trial by a shoulder tap from the experimenter. Between each trial, ear-defender headphones were worn to prevent extraneous auditory cues produced by movement of the board. Participants were instructed to produce mouth clicks and were allowed to click for as long as desired on each trial. It was estimated that participants usually clicked for between approximately 2 and 10 s before making a judgment.

### Procedures

Participants were blindfolded throughout the experiment, including from the point at which they entered the laboratory, so there were no visual cues available. The experiment started with a familiarization phase, followed by a distance learning task with feedback provided, followed by the main experiment which entailed an absolute distance task with no feedback. The familiarization phase provided participants with an opportunity to practice making mouth clicks, listening for the returning echoes, and using them to judge the distance of silent objects. At the start of the familiarization phase, participants completed an “object present or not” task, in which it was randomly decided for each trial whether no board was presented or the cork board was presented at a distance of 30 cm with no materials attached. The participant was instructed to click and report whether or not an object was present, with no time constraints. Each participant completed 10 trials with the board present and 10 trials with the board absent. The average score was 78% correct, and all participants stated that they were able to hear the difference in echoes when the board was present or absent.

Next, participants practiced judging distance using echolocation. On each trial, the uncovered cork board was positioned at one of three distances not tested in the main experiment, 15, 45, or 75 cm. Before each trial, the participant was informed of the object distance and was instructed to produce mouth clicks and to listen to the echoes. They were also asked pay attention to how the echoes changed across various distances across trials. Each test distance was presented 7 times, with 5 additional catch trials where the object was not presented (order randomized), giving 26 trials in total.

In the main experiment, participants performed an absolute distance echolocation task with no feedback. On each trial, an object was positioned at one of three distances (30, 60, or 90 cm) or was absent. The object distances were chosen from the three nearest test distances used by Tonelli et al. (2016). Tonelli et al. (2016) included two farther distances, but these were not used following pilot testing indicating that participants had considerable difficulty making distance judgments beyond the range tested. Participants were told that the object would be absent on some trials. Unlike the familiarization phase, participants were not informed of the object distance prior to producing echolocation clicks. Participants were instructed to produce mouth clicks and to report whether or not the object was present, and, if it was judged to be present, to report its distance in cm or mm. All participants reported judgments in cm only.

In a single experimental block, the object material (aluminum foil or foam) was kept constant, with 6 repetitions for each object distance, and 3 no-object catch trials. Thus, there were 21 trials per block, with order of presentation randomized. Measurements for the 2 types of object material with 2 repetitions for each block were tested in a single session (2 blocks x 2 materials x 21 trials = 84 trials in total). The order of presentation of the 4 blocks was randomized. The experiment lasted approximately 1 h.

### Statistical analyses

To investigate the accuracy of echolocation distance judgments, two measures of error were calculated. Firstly, the consistency (*C*) of each participant’s distance judgments was assessed for each test distance (*D*), by calculating *SD*/*D*, where *SD* is the standard deviation of judged distance for each *D*, across the twelve trials for a given *D* across the two test blocks. Larger values of *C* indicate lower consistency. The value of *C* for a given *D* is defined by

$$\:C=SD/D$$

Secondly, systematic error (*S*) was calculated. For a given *D*, the mean judged distance *J* was calculated across the 12 trials. *S* was calculated using the absolute value of log(*D*/*J*). Greater values of *S* indicate larger systematic errors. *S* is defined by:

$$\:S=\left|\text{log}\left(D/J\right)\right|$$

where |·| means the absolute value of ·.

Straight lines were fitted to the functions relating judged distance to actual distance, both expressed on log–log coordinates (Kolarik et al. 2017a). Slopes of these lines below 1 indicate a compressive relationship between judged and actual distance, and the lower the slope, the more compressive is the function. Repeated-measures *t*-tests were performed to compare the slopes for aluminum and foam objects. Repeated-measures analyses of variance (ANOVAs) were utilized to analyze how the logarithms of the distance judgments, consistency of distance judgments, and systematic error were influenced by material (aluminum, foam) and distance (30, 60, 90 cm). Where sphericity was violated, the Greenhouse-Geisser procedure was used to correct the degrees of freedom. The significance level was *p* < 0.05. Bonferroni correction was applied to post hoc analyses.

## Results

**Fig. 1:**
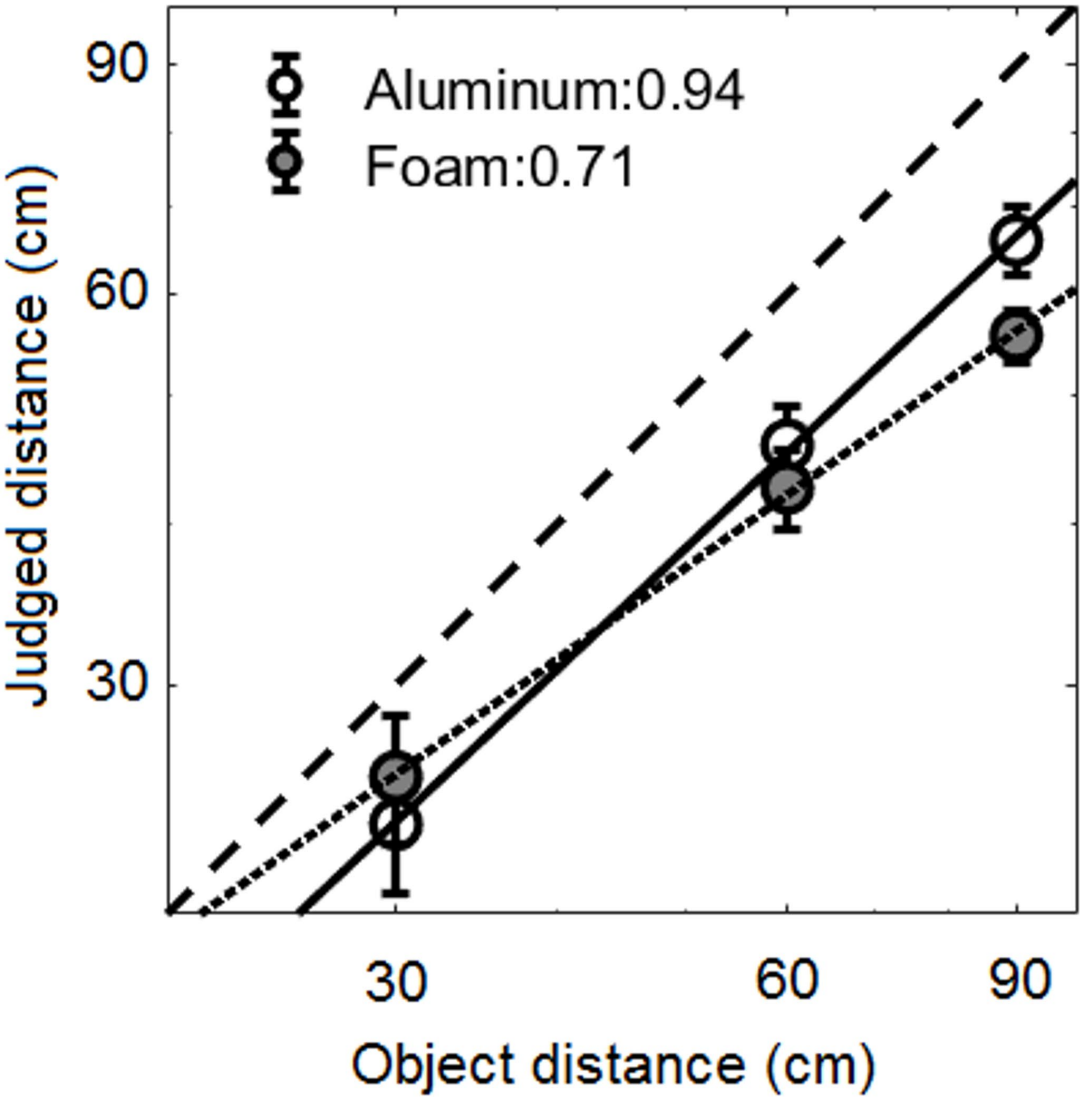
Mean echolocation absolute distance judgments as a function of object distance. Open and grey circles indicate data for aluminum and foam objects respectively. Error bars indicate ± 1 standard error across participants. Linear fits to the data for aluminum and foam on log-log coordinates are shown by solid and dotted lines, respectively, and the slopes are reported at the top of the panel. The dashed line indicates where the data would lie for perfect distance judgments

Figure 1 shows judged distance as a function of actual distance for aluminum (open circles joined by a solid line) and foam objects (grey circles joined by a dotted line), plotted on log–log coordinates. Distances were consistently underestimated for each test distance, and underestimation increased as object distance increased, more so for the foam object. The slope was significantly steeper for aluminum (0.94) than for foam (0.71) objects [*t*(10) = 2.68, *p* = 0.023]. This indicates that distance judgments were more compressed for foam than for aluminum objects.

A within-subjects repeated-measures ANOVA of the mean logarithmic distance judgments showed no main effect of material [*F*(1, 10) = 1.62, *p* = 0.231, η_p_^2^ = 0.14], but a significant main effect of distance [*F*(1.20, 11.95) = 72.80, *p* = 0.001, η_p_^2^ = 0.88], and a significant interaction between material and distance [*F*(1.25, 12.51) = 6.13, *p* = 0.023, η_p_^2^ = 0.38]. Post hoc tests indicated that distance estimates were significantly larger for aluminum objects than for foam objects for the farthest distance (90 cm) only.

Table 1 shows the percentage of object-present trials in which the object was reported (and an estimate of distance was given), and the percentage of false reports on object-absent catch trials. For object-present trials, the object was reported on the great majority of trials for both aluminum and foam objects, but there was a slight reduction in positive reports for the farthest distance (90 cm). For object-absent catch trials, the percentage of false reports of the object being present was higher for foam objects (45%) than for aluminum objects (18%).

**Table 1:** Summary of object-present reports and false reports. Mean and standard deviation of percentages of object-present trials for which the object was reported as present, and the percentage of false reports of the object being present for object-absent catch trials

% of object-present trials in which the object was reported (mean, SD)						% of false reports of object being present (mean, SD)
Object distance (cm)	30		60		90	*N*/A
Aluminum	100 (0)		100 (0)		95 (5)	18 (24)
Foam	100 (0)		100 (0)		98 (2)	45 (22)

**Fig. 2:**
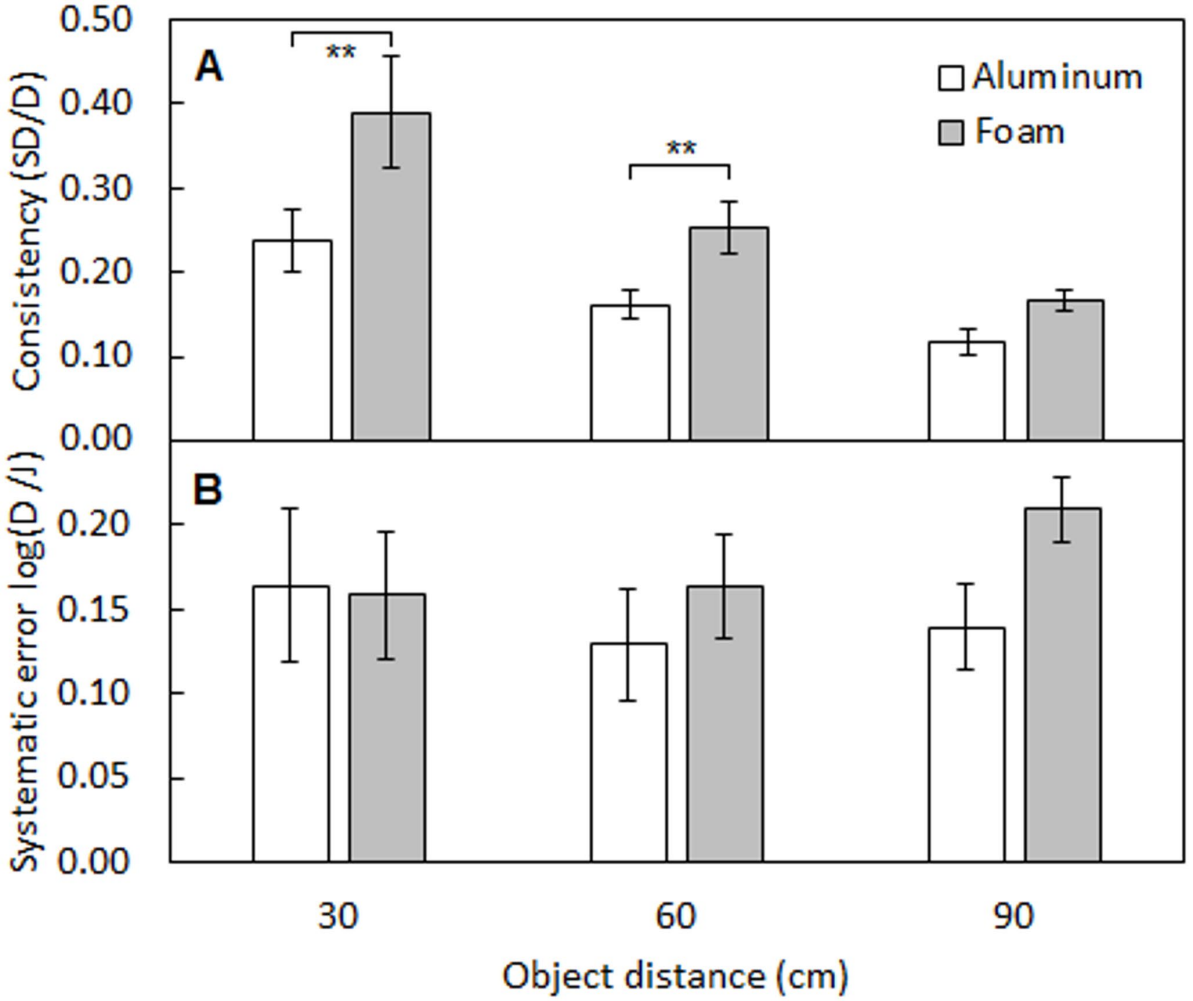
Mean consistency (*C*, panel A) and mean systematic errors (*S*, panel B) of distance judgments as a function of object distance. Open and grey bars indicate data for aluminum and foam objects, respectively. Error bars indicate ± 1 standard error across participants, and asterisks indicate significant differences, ** *p* < 0.01

Figure 2 panel A shows the mean consistency (*C*) of distance judgments for aluminum (open bars) and foam (grey bars) objects, for the three distances. *C* was highest (worst) for the closest distance and decreased as distance increased for both materials. At all object distances, judgments were more consistent for aluminum objects than for foam objects, the difference decreasing as object distance increased. A within-subjects ANOVA on the *C* values showed main effects of material [*F*(1, 10) = 13.64, *p* = 0.004, η_p_^2^ = 0.58] and distance [*F*(1.16, 11.61) = 9.38, *p* = 0.008, η_p_^2^ = 0.48], and a significant interaction between material and distance [*F*(2, 20) = 4.10, *p* = 0.032, η_p_^2^ = 0.29]. Post hoc tests with Bonferroni correction showed that *C* was significantly lower (better) for aluminum than for foam objects at 30 and 60 cm, but there was no significant difference for the distance of 90 cm.

Figure 2 panel B shows mean systematic errors, *S*, for aluminum (open bars) and foam (grey bars) objects, for each distance. A within-subjects ANOVA on the *S* values showed no significant main effects of material [*F*(1, 10) = 3.30, *p* = 0.099, η_p_^2^ = 0.25] or distance [*F*(1.31, 13.07) = 0.50, *p* = 0.540, η_p_^2^ = 0.048] and no significant interaction between material and distance [*F*(2, 20) = 3.41, *p* = 0.053, η_p_^2^ = 0.25], although there was a trend for *S* to be higher (worse) for the foam than for the aluminum for the farthest distance.

## Discussion

The results showed that, after a small amount of training, blindfolded, sighted participants were able to use echolocation to judge the distance of silent objects. The hypotheses were partially supported by the findings. The data relating judged to actual distance were well fitted by compressive power functions, similar to what has been found for sound-producing sources (Zahorik et al. 2005), as predicted. The compression was greater for foam than for aluminum objects. Judged distance was significantly greater (and closer to actual distance) for the aluminum object than for the foam object for the farthest test distance only. The consistency, *C*, of distance judgments was better for aluminum than for foam for the two closest test distances only. Systematic errors, *S*, did not change significantly with distance, consistent with results reported by Tonelli et al. (2016), or object material. The results suggest that for the object materials tested in the current study, material affects the consistency of echolocation judgments.

The slopes of the distance estimates observed for echolocation (0.94 and 0.71 for aluminum and foam respectively) were similar to or steeper than slopes obtained for distance estimates for sound-producing sources reported in previous studies. For normally sighted and normally hearing listeners judging the distance of sounds in virtual space, Zahorik (2002a) reported an average slope of 0.39 for speech and noise sources and Kolarik et al. (2017a) reported slopes ranging between 0.58 and 0.82 for speech, music, and noise sources. In Zahorik’s (2005) review of 84 data sets of distance judgments, the mean slope was 0.54, and none of the data sets had a slope over 0.9. We are unaware of any studies of distance estimates that report a slope greater than 1 (which would indicate expansion of the internal representation of auditory space) for sound-producing objects. Taken together, the current results and those from the literature suggest a general tendency for compression of auditory distance estimates to occur, but the compression is smaller for judgments based on echolocation than for judgments of sound-producing objects. It may be the case that when the cues do not change with distance (near or above the “auditory horizon”), the mean distance judgment does not vary with distance, leading to compression. However, distance judgements are likely to be largely a function of the utility of the available cues, which is probably why not much compression was observed in the case of aluminum object in the current study, due to the high reflectivity of the material.

It is possible that our participants relied on discrimination strategies rather than absolute judgments: given the familiarization task, participants might have deduced that only three test distances were used. While the possibility that discrimination strategies were used rather than absolute judgments cannot be discounted, the consistency (*C*) of distance estimates suggests that this is unlikely. As shown in the upper panel of Fig. 2, distance estimates were least consistent for the closest object distance, and became more consistent as object distance increased, especially for foam objects. This suggests that participants were not simply relying on a “nearer, mid-distance, farther” strategy with anchored estimates for three distances, for which similar consistency would be observed across the three test distances.

*C* was poorest for the nearest objects, for which the echoes would have been the most salient. The lowest consistency for echolocating objects closest to the participants was also reported by Tonelli et al. (2016) for participants tested in an anechoic chamber. The authors suggested this may have been partially due to the location of the self-generated finger-snaps used for echolocation interfering with the participant’s egocentric frame of reference, which would likely be centered on the head. Another possibility is that, in the current experiment as well as that of Tonelli et al. (2016), the poorer consistency for the closer distances may have resulted from slight movement of the participants, such that the object distance on a given trial was jittered around its nominal value. For close distances, small changes in distance resulted in relatively large changes in the available cues, so the small jitter in distance would have resulted in large changes in judged distance and hence large values of *C*.

Consistent with the proposal of Larsen et al. (2008), it is possible that, beyond a certain distance, all sounds are judged as being at about the same distance, because the acoustic cues to distance hardly change with distance. For our stimuli, the echoes were probably only just audible for the farthest distance, and this may have led each participant to give roughly the same estimate of distance for a given material whenever they could only just hear the echo. Also, it may be easier to judge when an echo is only just audible than to discriminate changes in the level of clearly audible echoes (as would be the case for closer distances). Future work could use a wider range of distances to confirm the existence of an “auditory horizon” for echolocation judgments of silent objects, similar to the limits for judging the distance of sound-producing objects (Bronkhorst and Houtgast 1999).

Further work could also investigate whether extended echolocation training leads to more consistent judgments for closer distances for different object materials, and assess how much training is required for significant improvements to be observed. Norman et al. (2021) showed that a 10-week training program used with normally sighted and blind individuals led to significant improvements for echolocation tasks including size discrimination, orientation perception, and virtual and natural environment navigation. The effects of training on echolocation-based absolute distance judgments and on measures of consistency and systematic error have yet to be assessed. The current findings suggest that a focus on training to improve consistency for closer object distances may be beneficial for spatial awareness when echolocating.

A limitation of the current experiment is that it tested only younger blindfolded, sighted individuals over a limited range of object distances and materials. It is unknown if the findings will generalize to individuals outside this group, such as older individuals with hearing loss. Akeroyd et al. (2007) showed that distance discrimination for sound-producing sources among hearing-impaired participants was significantly degraded when reverberation cues only were available. Hearing loss in older individuals may make it more difficult to use echoic cues for distance estimation. This requires experimental testing. Another limitation of the present work is that only three distances were tested, making it hard to be sure that distance judged using echoes from self-generated clicks really is a power function of actual distance. Distance judgements using additional object distances would enable better characterization of the function relating judged to actual distance.

A further limitation is that the objects always directly faced the participant and testing was conducted only in a single low-reverberation room. Future studies could compare absolute distance judgments for objects presented at a range of angles relative to the participant using a range of acoustic environments and testing fully blind and older individuals as well as young normally hearing individuals. Investigation of the effectiveness of echolocation cues in isolation for absolute distance estimation could be undertaken by recording echolocation clicks and manipulating them to restrict available cues such as spectral coloration and level (Schenkman and Nilsson 2011). Blind individuals have been reported to show significantly higher detection accuracy (Schenkman and Nilsson 2010, 2011) and be able to detect objects using echolocation at greater distances (Kolarik et al. 2017b) than normally sighted individuals. Blind individuals have also been shown to have greater sensitivity to sound echoes (Dufour et al. 2005), which may lead to enhanced accuracy and consistency for absolute echolocation distance estimates, as well as the ability to detect less reflective objects (such as the foam object tested in the current study) at greater distances than for sighted participants. However, this needs to be confirmed. Future work could also assess if blind individuals have more confidence in their echolocation distance judgments, and which percepts participants feel are similar across different materials, which may provide insight regarding the limits of echolocation for distinguishing between objects of different materials (Hausfeld et al. 1982). Different response modes could also be assessed, such as moving to the perceived location of the object. Such estimates could be compared to those for verbal responses, as were used in the current study. For a discussion of the issues of how response mode for behavioral measurement can affect localization performance, see Kolarik and Moore (2025). The present findings are consistent with the idea that compression of judged distance occurs in most cases where auditory information is utilized to construct internal spatial maps, although the measured compression for the aluminum board was small. Further evidence is needed from studies that map novel auditory information to internal representations of distance, before strong claims can be made.

In summary, estimates of distance to an object made using a novel echolocation skill were related to actual distance by a compressive power function. For the less reflective foam object, the compression was greater (farther distances were underestimated to a greater extent), and responses were more variable (i.e. less consistent). Taken together with work on the judged distance of sound-producing objects (Zahorik et al. 2005), the findings suggest that compression may be a general characteristic of auditory distance judgments, although the amount of compression found here was less than typically reported for distance judgments of sound-producing objects. An implication of the results is that blind people being trained to use echolocation for the judging distances of objects should be made aware of the tendency for the perceived distance of farther objects to be underestimated, especially for less reflective materials.

## Data Availability

Data is available at https://osf.io/twz52/?view_only=fde797e12c8e419e8c2562c1d8ab61c9.
